# Projected climate change threatens pollinators and crop production in Brazil

**DOI:** 10.1371/journal.pone.0182274

**Published:** 2017-08-09

**Authors:** Tereza Cristina Giannini, Wilian França Costa, Guaraci Duran Cordeiro, Vera Lucia Imperatriz-Fonseca, Antonio Mauro Saraiva, Jacobus Biesmeijer, Lucas Alejandro Garibaldi

**Affiliations:** 1 Instituto Tecnológico Vale Desenvolvimento Sustentável, Belém, Pará, Brazil; 2 Escola Politécnica da Universidade de São Paulo, São Paulo, São Paulo, Brazil; 3 Instituto de Biociências da Universidade de São Paulo, São Paulo, São Paulo, Brazil; 4 Naturalis Biodiversity Center, Leiden, Netherlands; 5 Instituto de Investigaciones en Recursos Naturales, Agroecología y Desarrollo Rural (IRNAD), Sede Andina, Universidad Nacional de Río Negro (UNRN) and Consejo Nacional de Investigaciones Científicas y Técnicas (CONICET), San Carlos de Bariloche, Río Negro, Argentina; Indian Institute of Science, INDIA

## Abstract

Animal pollination can impact food security since many crops depend on pollinators to produce fruits and seeds. However, the effects of projected climate change on crop pollinators and therefore on crop production are still unclear, especially for wild pollinators and aggregate community responses. Using species distributional modeling, we assessed the effects of climate change on the geographic distribution of 95 pollinator species of 13 Brazilian crops, and we estimated their relative impacts on crop production. We described these effects at the municipality level, and we assessed the crops that were grown, the gross production volume of these crops, the total crop production value, and the number of inhabitants. Overall, considering all crop species, we found that the projected climate change will reduce the probability of pollinator occurrence by almost 0.13 by 2050. Our models predict that almost 90% of the municipalities analyzed will face species loss. Decreases in the pollinator occurrence probability varied from 0.08 (persimmon) to 0.25 (tomato) and will potentially affect 9% (mandarin) to 100% (sunflower) of the municipalities that produce each crop. Municipalities in central and southern Brazil will potentially face relatively large impacts on crop production due to pollinator loss. In contrast, some municipalities in northern Brazil, particularly in the northwestern Amazon, could potentially benefit from climate change because pollinators of some crops may increase. The decline in the probability of pollinator occurrence is found in a large number of municipalities with the lowest GDP and will also likely affect some places where crop production is high (20% to 90% of the GDP) and where the number of inhabitants is also high (more than 6 million people). Our study highlights key municipalities where crops are economically important and where pollinators will potentially face the worst conditions due to climate change. However, pollinators may be able to find new suitable areas that have the potential to improve crop production. The results shown here could guide policy decisions for adapting to climate change and for preventing the loss of pollinator species and crop production.

## Introduction

One of the key challenges addressed by the World Summit on Food Security is the necessity for countries to properly address the impact of climate change in order to achieve food security [1]. According to the FAO (Food and Agriculture Organization), food security exists when all people, at all times, have physical, social and economic access to address their dietary needs and food preferences for an active and healthy life [1]. Food security can be affected by climate change because it may change crop growth and production [2], impacting crop price and the food market and exacerbating hunger, land abandonment, migration and urbanization [3]. At the global scale, climate change is expected to lead to a 14% decline in per capita cereal production by 2030 [4], particularly affecting tropical areas [5]. In Africa and South Asia, 8% yield losses are expected across all crops by 2050 [6], with developing countries being more vulnerable [7], potentially enhancing the decline in crop productivity, particularly in countries that currently have a high prevalence of hunger [2]. Brazilian agricultural production is also expected to be affected by climate change. Between 2 and 5 billion US$ is the projected loss to be suffered by 2070, with coffee-growing areas showing a 30% decrease in the southeastern region [8].

An additional challenge to agriculture related to climate change is the loss of crop pollinators, with pollination being an ecosystem service that is important to maintain the production of the majority of crops [9]. Crops have different degrees of dependency on animal pollinators, and a global evaluation showed that 85% (91 of 107 crops) are pollinator dependent to some degree [10]. In Brazil, 60% of crops (85 of 141) are pollinator dependent, with another 39 crops that do not depend on animal pollination and 17 crops lacking data [11]. The area cultivated with pollinator-dependent crops has increased in recent decades [12], intensifying the need for pollinators and pollination. In addition, pollinator-dependent crops are important for human diet as a main source of micronutrients, such as vitamins A and C, calcium and folic acid [13], and a geographical equivalence was found between areas with a high vitamin A deficiency and pollinator-dependent crops that produce such vitamins [14]. These findings highlight the challenges for intensifying research on crop pollinator species and their interactions, emphasizing the urgent need for further research [15].

Climate change is ongoing, involving changes in precipitation and temperature regimes. According to the Intergovernmental Panel on Climate Change (IPCC), climate change will potentially lead to an average temperature increase of 2°C to 4°C by 2050 depending on the emission scenario [16]. Declines in pollinators as a result of climate change have already been suggested for honey bees [17] and bumblebees [18]. Other species have also shifted their distribution toward the poles [19, 20] or to higher elevations [21] or have exhibited more complex responses [22], seeking milder habitat conditions. Climate change is also affecting the interaction between species [23], changing the structure of interaction networks [24], resulting in changes in phenological synchronization [25] and leading to mismatches in the geographic distribution of interacting species [26]. In addition, under climate change, the biota tends to show homogenization, with generalist species, which usually have broader abiotic requirements, becoming more prevalent [27], since species with narrow ecological niches or habitat preferences are likely to disappear [28].

The objective of this paper is to assess the impact of projected climate change on the geographic distribution of crop pollinators of 13 Brazilian crops and to estimate its relative impact on crop production. To this end, we analyzed pollinator shifts due to climate change for each crop using species distribution modeling. We evaluated the impact of climate change in each municipality where these crops are produced as well as the total crop production, the gross domestic product (GDP) per municipality and the number of inhabitants.

## Materials and methods

The main pollinators of 13 Brazilian crops analyzed here were defined in another study, totaling 95 species [29] (S1 Table). The total number of Brazilian crops is still unknown, with most regional fruits and vegetables being produced by only local farmers. A recent assessment evaluated the pollinator dependency of 141 crops, but this is not the total number of Brazilian crops [11]. Moreover, there is a lack of information about the pollinator dependency of crops as well a lack of knowledge related to the main crop pollinators [11, 29]. Additionally, the values of annual production per municipality are not available in the public data repository for all crops (Brazilian Institute of Geography and Statistics—IBGE), and such values are necessary for the estimations proposed here. Thus, this study analyzes 13 crops for which we have already determined the pollinator dependency and the main pollinators and for which we have the values of annual production per Brazilian municipality.

We assessed the impact of projected climate change on crop pollinators using species distribution modeling (SDM), a computational technique that determines potential areas of species occurrences and forecasts their future distribution [30]. We retrieved information about the occurrence points of each pollinator species from the speciesLink data portal (Centro de Referência em Informação Ambiental, CRIA) and from the Global Biodiversity Information Facility (GBIF) data portal. Both repositories contain biodiversity data deposited mainly in biological collections and museums (S2 Table).

In addition to the occurrence points, SDM uses environmental variables to determine suitable areas for species potential distribution. We used climatic variables obtained from Worldclim [31] with a resolution of 5 arc-minutes (approximately 10 x 10 km cell size at the Equator). From the 20 variables available under current climatic conditions, we calculated the nine least correlated variables [32]: altitude, mean diurnal range, isothermality, mean temperature of the driest quarter, annual precipitation, precipitation of the driest month, precipitation seasonality, precipitation of the warmest quarter, and precipitation of the coldest quarter. We used the same variables to forecast the future potential distribution projected by the Met Office Hadley Centre (HadGEM2-CC) and the University of Tokyo and collaborators (MIROC-ESM-CHEM) for the year 2050. We built the ensemble forecasting of both projections, which consists of a weighted sum scheme for merging the final models obtained [33]. We used the representative concentration pathways (RCP) set to 8.5, which specifies a likely global mean surface temperature increase by the end of the 21st century of 2.6°C to 4.8°C [16]. This scenario was chosen for projecting the greatest increase in the emission and, consequently, the most pronounced changes. For conservation purposes, it is important to detect the areas that are most suitable, even in more extreme scenarios, minimizing costs and maximizing the chance of effectively protecting the species. This approach was used in previous studies [34, 35, 36].

The Maximum Entropy algorithm (Maxent) [37] was used to perform the SDM. This algorithm is particularly useful because it can be applied to datasets using only presence records of species (as opposed to other algorithms that require absence data) [38]. We used the area under the curve (AUC) of the receiver-operator graph to estimate the accuracy of the modeling process in a test data set (20% of the total occurrence data) [39].

We analyzed the distribution of the pollinators of 13 Brazilian crops (S1 Table), with different dependencies for animal pollination: essential for pollination (acerola, annatto, passion fruit); great dependency (avocado, guava, sunflower, tomato); modest dependency (coconut, coffee, cotton); and little dependency (bean, mandarin, persimmon) (dependencies according to [29]). The values of annual production (tons) per crop were retrieved from the Brazilian Institute of Geography and Statistics (IBGE) website per municipality (year 2013, except for acerola, for which the figures correspond to 2006).

To evaluate the impact of projected climate change on pollinators in the Brazilian municipalities that produce a particular crop, we merged all models obtained for all pollinator species for each crop considering current conditions and did the same considering future forecasts (the ensemble of the Had and MIROC scenarios). This procedure resulted in one model for the potential distribution of all pollinators under current conditions and one model for the future conditions for each crop. In the subsequent step, we subtracted the values of the future potential distribution (represented as the occurrence probability) from the current one, also per crop. This final model represents the potential shift in pollinator occurrence per pixel and expresses an index that varies from -1 (100% decrease in pollinator occurrence, i.e., no pollinator will occur in that particular pixel) to +1 (100% increase in pollinator occurrence, i.e., all pollinators will occur in that particular pixel).

In the first step, we aimed to evaluate the impact of projected climate change on pollinators considering all of the abovementioned crops. To this end, we calculated the average potential shift in pollinator occurrence for the whole country. Moreover, we calculated the number of municipalities where each crop is produced that will potentially face an increase or decrease in pollinator occurrence considering the following: i) the GDP (the monetary value of all of the finished goods and services produced within a country's borders in a specific time period) per municipality, ii) the percentage of that particular crop in the GDP per municipality, and iii) the population (number of inhabitants). The value of the GDP and the population per Brazilian municipality were also retrieved from the IBGE website (year 2013).

In the next step, the potential shift in pollinator occurrence was plotted for the Brazilian municipalities where the particular crop is produced. For this step, we standardized the final scale to vary from -1 (representing the highest value of production and the highest decrease in pollinator occurrence probability) to +1 (representing the highest value of production and the highest increase in pollinator occurrence probability) to facilitate the interpretation of the results.

All the procedures were performed using Postgres (The PostgreSQL Global Development Group) for database management, the ‘raster’ package [40] of R (the R Project for Statistical Computing), and QGIS (Open Source Geospatial Foundation Project).

## Results

**Fig 1:**
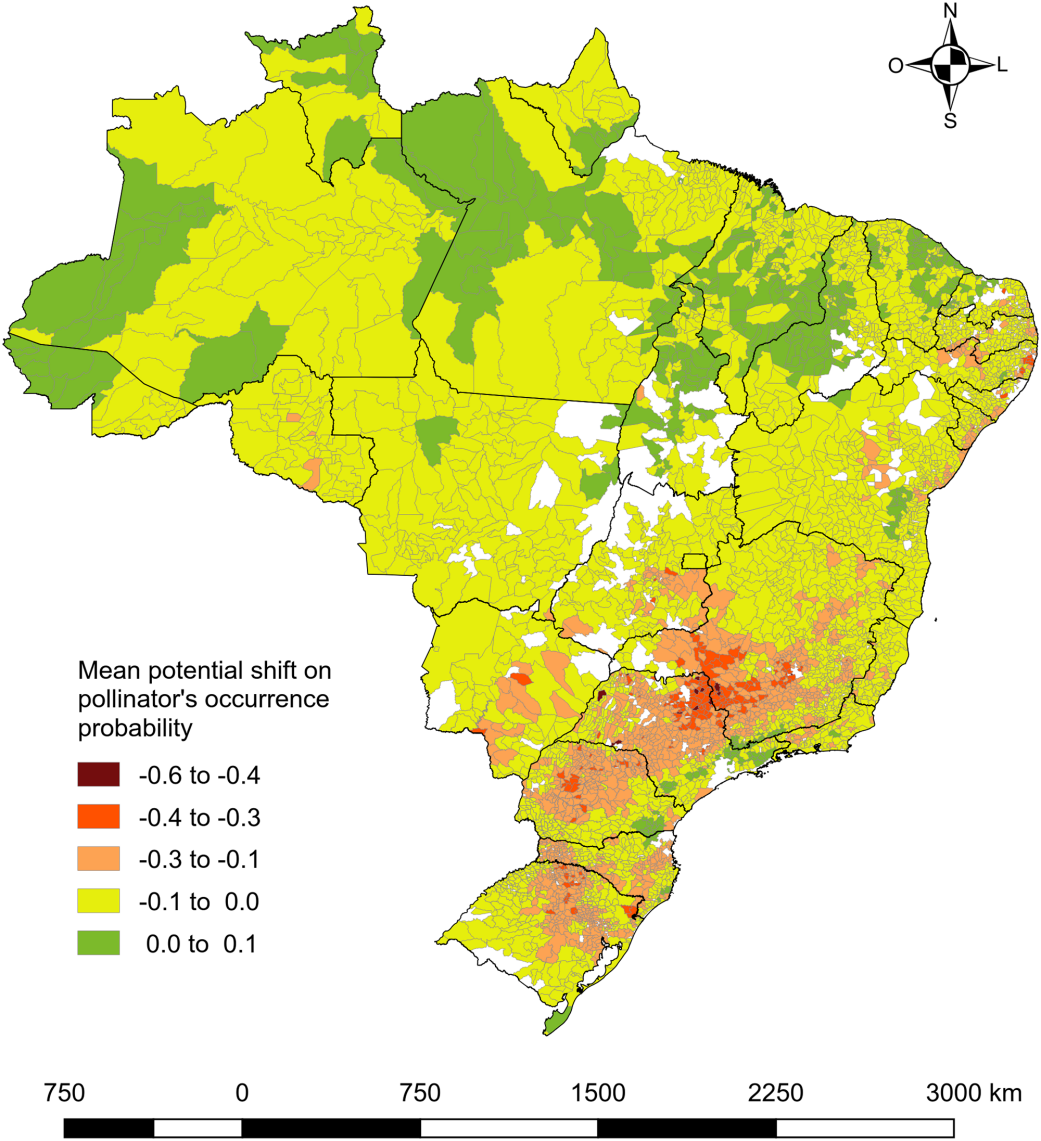
Mean potential shift in the pollinator occurrence probability related to projected climate change for 2050 in the Brazilian municipalities where the 13 analyzed crops are produced. Values vary from -1 (decrease of 100% in pollinator occurrence probability; red to yellow) to +1 (increase of 100%; green to blue). Blank areas correspond to the municipalities where there is no production of the analyzed crops.

The resulting mean potential shift in the pollinator occurrence probability for all analyzed crops (13 crops) shows that projected climate change will likely affect pollinators differently in different regions of Brazil (Fig 1). A slight decline in the probability of pollinator occurrence could occur in most areas (Fig 1—yellow color). A larger decline could occur mostly in the southern areas (orange and red colors). However, some areas, especially in the northern region, will likely experience a small increase in the probability of pollinator occurrence (green color), indicating an increase in the suitability of these regions. Considering all 13 crops, an overall 0.13 (SD = 0.11) average decrease in the pollinator occurrence probability was found as well as a high percentage of municipalities (88%) that will potentially face a decrease in the pollinator occurrence probability, contrasting with a 12% increase (S3 Table).

**Fig 2:**
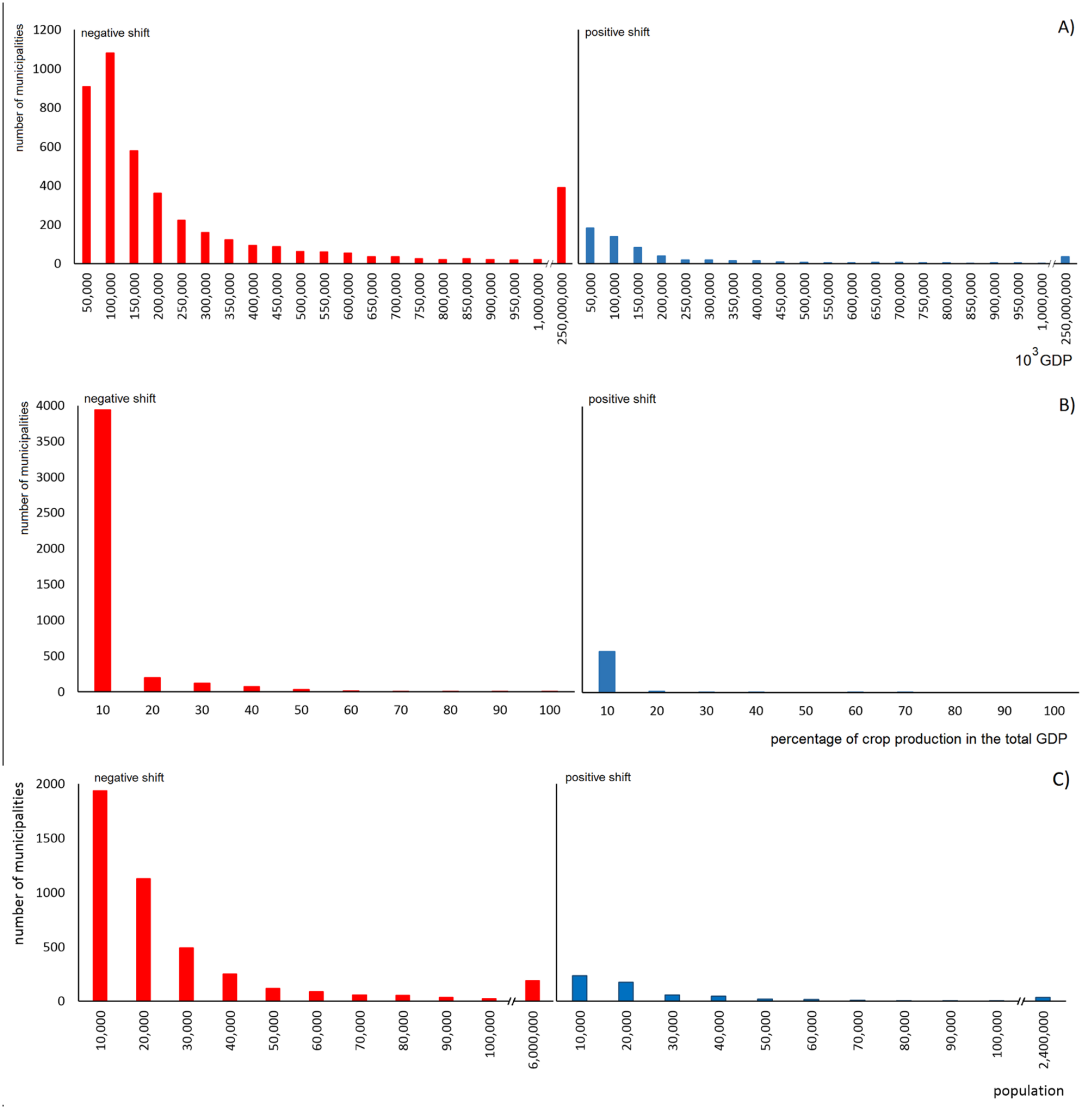
A) Frequency of municipalities that will face potential negative and positive shifts in pollinator occurrence probability considering the gross domestic product (GDP); B) the percentage of the production of the analyzed crops in the total GDP (acerola was not included due to the lack of data); and C) population.

Most municipalities will likely face a decline in the probability of pollinator occurrence and also have the lowest GDP (less than R$1bi) (Fig 2A). Few municipalities will potentially face an increase in probability, also presenting low values of GDP (Fig 2A). However, almost 400 municipalities with higher GDP values (between R$1bi and R$250bi) will face a decline in the probability of pollinator occurrence (Fig 2A). Moreover, for most of the municipalities that will likely experience a decline in pollinator occurrence, crop production represents 10% of the GDP; however, for others, crop production represents higher numbers (20% to 90% of the GDP) (Fig 2B). The percentage of crop production in the total GDP is also low (less than 10%) in the few municipalities that will likely face positive shifts (Fig 2B). A decline in the probability of pollinator occurrence also correlates with most of the municipalities with fewer than 50,000 inhabitants but also includes some municipalities with the highest number of inhabitants (6 million people) (Fig 2C). However, positive potential shifts were also found in small municipalities (fewer than 40,000 inhabitants) as well as some populous municipalities (2.4 million people).

**Fig 3:**
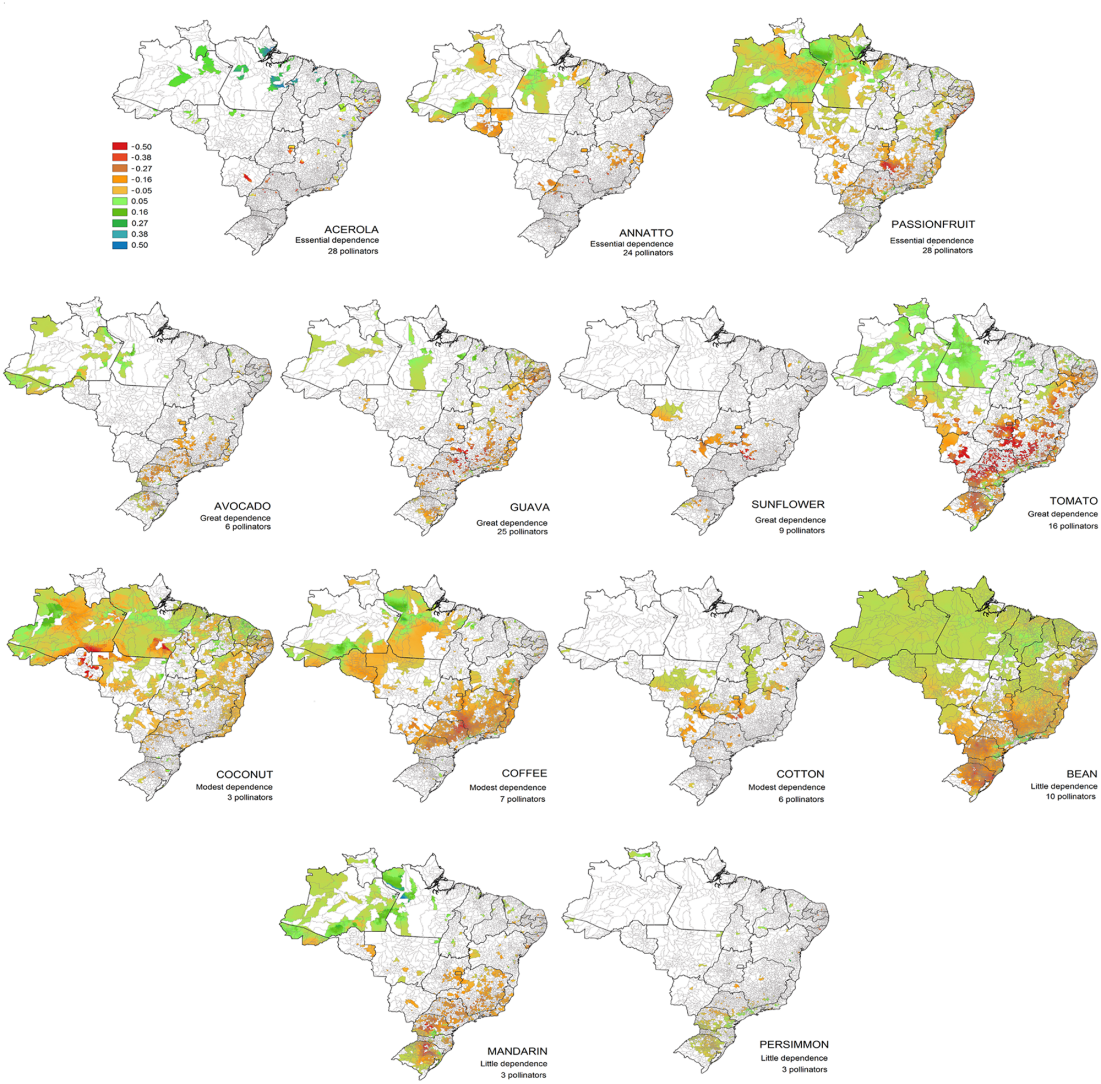
Potential shift in the pollinator occurrence probability related to projected climate change for 2050 in the Brazilian municipalities where each crop is produced. Values vary from -1 (decrease of 100% in pollinator occurrence probability; red to yellow) to +1 (increase of 100%; green to blue). Crops have different levels of dependence on animal pollination (according to Giannini et al. 2015b). The list of pollinators for each crop can be found in S1 Table.

The potential shift in pollinator occurrence probability related to projected climate change in each Brazilian municipality varies strongly, as does the pattern for the different crops (Fig 3). The decline in the occurrence probability of the pollinators for each crop varies from 2% (persimmon) to 25% (tomato) (Table 1A) and can potentially affect from 9% (for mandarin) to 100% (sunflower) of municipalities where each crop is produced (Table 1B). Most of the suitable future areas can be found in the northern areas, where almost all crops are produced (except sunflower and cotton). Some pollinators will also likely find potentially suitable areas in the southern areas of Brazil, where avocado, sunflower, tomato, bean, mandarin and persimmon are produced. However, most of the eastern region of Brazil, where many crops are currently produced, will likely experience a decrease in pollinator occurrence probability (except for persimmon). The areas where guava, tomato and coffee are produced will likely face the highest decrease in pollinators. Based on the shift in the pollinator occurrence probability and the crop production of each municipality, our results show that some municipalities with the highest value of production will potentially face important decreases in the pollinator occurrence probability (list of municipalities in S4 Table).

**Table 1:** Shifts for each crop analyzed considering A) the decrease and B) the increase in the pollinator occurrence probability and the number of municipalities potentially affected (scientific name of each crop can be found in S1 Table).

	Occurrence probability %	Occurrence probability STD	Number of municipalities	Total number of municipalities that produce each crop	%
**A) Decrease in pollinator occurrence probability**					
acerola	14.0	10.2	150	201	74.6
annatto	14.7	8.7	220	258	85.3
avocado	10.4	7.3	613	673	91.1
bean	9.9	8.8	3527	4188	84.2
coconut	5.6	4.4	1522	1753	86.8
coffee	15.3	8.6	1631	1708	95.5
cotton	6.9	6.3	318	321	99.1
guava	16.0	11.1	793	838	94.6
mandarin	15.1	9.8	1263	13645	9.3
passion fruit	10.8	9.9	973	1155	84.2
persimmon	2.8	2.2	503	579	86.9
sunflower	17.7	15.6	101	101	100.0
tomato	25.5	15.4	1521	1743	87.3
**B) Increase in pollinator occurrence probability**					
acerola	4.8	5.2	51	201	25.4
annatto	2.5	2.0	38	258	14.7
avocado	4.4	4.8	60	673	8.9
bean	1.7	2.2	661	4188	15.8
coconut	2.8	2.9	231	1753	13.2
coffee	4.8	4.7	77	1708	4.5
cotton	20.4	13.7	3	321	0.9
guava	3.1	3.8	45	838	5.4
mandarin	3.8	4.2	82	1345	6.1
passion fruit	7.3	9.0	182	1155	15.8
persimmon	3.5	2.5	76	579	13.1
sunflower	0.0	0.0	0	101	0.0
tomato	4.3	3.1	222	1743	12.7

## Discussion

Considering the importance of pollination in crop production, we investigated the potential impact of climate change on the distribution of pollinator bees for some Brazilian crops. We found that the pollinator occurrence probability will decrease in most of Brazil by the year 2050. The highest decrease will potentially be found in the southern areas. In contrast, some northern areas will show a slight increase in the occurrence probability of crop pollinators.

The predicted effect of climate change will potentially reduce the occurrence of pollinators in most of the municipalities with the lowest GDP. This finding has important implications since it is expected that the effects of climate change will also cause a decline in crop productivity independent of the pollination deficits [8, 2] or, additionally, change the dependence of crops on pollinators due to heat wave increases, as already discussed [41]. These joint effects could bring additional reductions in agricultural income in already poor municipalities, reinforcing their socioeconomic vulnerability. Worryingly, the highest decline in the probability of pollinator occurrence is projected to occur in the majority of municipalities (4000 municipalities) in which crop production accounts for the lowest percentage of the total GDP (10%), allowing few possibilities for future agricultural expansion if those areas with currently low production face a future reduction in pollinators. These cities are mainly found in the central and southern areas of Brazil. Municipalities with the highest GDP will also face a reduction in the occurrence of pollinators. However, the socioeconomic impact will be lower since the GDP is not so highly dependent on crop production. Considering this scenario, it is important to delineate strategies aiming to reduce the deficit of crop pollination and, at the same time, enhance crop productivity, promoting a better income for crop producers and helping to minimize further losses of natural areas for agriculture [42].

When analyzing each crop separately, we found that guava, tomato, coffee and mandarin pollinators will potentially be the most affected by pollinator loss. Of these, guava and tomato are greatly dependent on pollinators and may be greatly affected. Specifically, considering the impact on the pollinators of tomato, our results are corroborated by a previous study that analyzed five pollinator species of tomato in Brazil, showing reductions of between 10 and 70% in their distributional range [43]. Although coffee is modestly dependent on pollinators, it is mostly produced in the southeastern region (the most affected region according to our scenario) with very high values of annual production and the second highest economic value of pollination in Brazil (almost US$ 2 billion/year) [11]. Mandarin has little dependence on pollinators but is also highly produced in the southern areas of Brazil. Therefore, pollinator loss for these crops may cause high economic and social impacts. Interestingly, new suitable areas for the production of the abovementioned crops may be found in the northern regions, and the feasibility of increasing their cultivation in these regions should be investigated. Particularly in western Amazonia (northern Brazil), there are continuous areas of natural habitats, and many crops are still collected in an extractive manner [44, 45] or produced on small farms. In contrast, in the southern region of Brazil, natural areas are fragmented and croplands are usually more extensive and homogeneous. Additionally, new habitat losses will likely continue to occur all over the country, and there is a debate in the literature regarding whether the necessity of expanding agricultural areas to compensate the decrease in crop yield due to climate change will increase [46], which could bring new challenges to pollinator protection. All of the abovementioned aspects are intricately associated and can affect agricultural production.

Future work should consider the impact of climate change on other aspects of Brazilian crop production, such as the reduction or deficiency in the availability of water for irrigation and possible phenology mismatches between flowering and pollinators. Moreover, the impact of climate change on the Brazilian crop itself should be taken into account since production is highly dependent on precipitation and temperature regimes. In addition, new studies could consider the effect of land use changes on agricultural areas in addition to climate. Moreover, climate change can potentially impact pollinator species in other ways, for example, changing the size of their populations, which could have additional impacts on Brazilian crop productivity. Another key factor is related to the quality of the data. More work is necessary to fill knowledge gaps about crops' effective pollinators and animal pollination dependence as well as data on annual crop production on Brazil. Notably, there are few data available about regional crops, whose production is often based on family farming and that are sources of income for the local economy.

Here, we propose a comprehensive methodology to analyze the impact of climate change on crop pollinators based on species distribution modeling that can be easily applied to other data, crops or regions. We show that climate change can affect pollinator species differently and that the relative impact on crop production needs to be considered when planning strategies that involve food production for medium- to long-term periods. Conservation strategies for crop pollinators are urgent and deepen the discussions about climate refuges and pollinator-friendly agricultural practices. Biodiversity data involving crop pollinators and production need to be gathered and shared to improve the accuracy of analyses that can ultimately be translated into effective strategies to guarantee the ecosystem services delivered by pollinator species.

## Supporting information

S1 Table Pollinator species of each agricultural crop analyzed. Each crop presents different dependence for animal pollination (Giannini et al. 2015b). Pollinator species used here were previously considered as being effective, occasional or potential pollinators of each analyzed crop (Giannini et al. 2015a).
(DOCX)

S2 Table Data sources retrieved from speciesLink and GBIF website. (DOCX)

S3 Table Shifts on pollinators’ occurrence probability for all crops analyzed (13 crops) considering A) the overall average of decrease in probability; B) the number and percentage of municipalities that will potentially face decrease or increase on pollinators’ occurrence probability (total number of municipalities analyzed equals to 4975). (DOCX)

S4 Table Municipalities that will potentially face the highest negative shift on pollinators’ occurrence probability and that present the highest percentage of gross domestic product (GDP) associated to the analyzed crops. We considered 25% of the highest values of negative shift on pollinators and, from those, the 15 municipalities that present the highest percentage of GDP associated to the analyzed crop. Acerola was not included due to the lack of data.
(DOCX)
