# Supplementary material for: Projected climate change threatens pollinators and crop production in Brazil
Source: PLoS One. 2017 Aug 9;12(8):e0182274. doi: 10.1371/journal.pone.0182274 (PMC5549956; doi:10.1371/journal.pone.0182274)
Supplement: S1 Table — Each crop presents different dependence for animal pollination (Giannini et al. 2015b). Pollinator species used here were previously considered as being effective, occasional or potential pollinators of each analyzed crop (Giannini et al. 2015a). (DOCX) [file pone.0182274.s001.docx]

**S1Table.** Pollinator species of each agricultural crop analyzed. Each crop presents different dependence for animal pollination (Giannini et al. 2015b). Pollinator species used here were previously considered as being effective, occasional or potential pollinators of each analyzed crop (Giannini et al. 2015a).

| **Crop** | **Pollinator** |
| --- | --- |
| **ESSENTIAL DEPENDENCE FOR ANIMAL POLLINATION** | |
| **Annatto** *(Bixa orellana)* | *Bombus morio; Bombus transversalis; Centris aenea; Centris obsoleta; Cephalotrigona capitata; Epicharis affinis; Epicharis flava; Epicharis rustica; Eulaema cingulata; Eulaema meriana; Eulaema nigrita; Exomalopsis analis; Exomalopsis auropilosa; Melipona fasciculata; Melipona melanoventer; Melipona quadrifasciata; Melipona subnitida; Oxaea lavescens; Schwarziana quadripunctata; Tetragonisca angustula; Trigona spinipes; Xylocopa aurulenta; Xylocopa frontalis; Xylocopa grisescens; Xylocopa muscaria* |
| **Acerola** (*Malpighia emarginata, M. glabra)* | *Centris aenea; Centris caxiensis; Centris denudans; Centris flavifrons; Centris fuscata; Centris longimana; Centris maranhensis; Centris mocsaryi; Centris nitens; Centris obsoleta; Centris rhodoprocta; Centris scopipes; Centris spilopoda; Centris sponsa; Centris tarsata; Centris trigonoides; Centris varia; Centris vittata; Epicharis affinis; Epicharis albofasciata; Epicharis analis; Epicharis bicolor; Epicharis cockerelli; Epicharis flava; Epicharis xanthogastra; Melipona quadrifasciata; Nannotrigona testaceicornis; Partamona cupira; Tetragonisca angustula; Trigona spinipes* |
| **Passionfruit** (*Passiflora edulis, P. alata)* | *Acanthopus excellens; Bombus morio; Bombus pauloensis; Centris denudans; Centris flavifrons; Centris longimana; Centris lutea; Centris scopipes; Centris sponsa; Epicharis analis; Epicharis bicolor; Epicharis fasciata; Epicharis flava; Epicharis nigrita; Eufriesea surinamensis; Eulaema bombiformis; Eulaema cingulata; Exaerete smaragdina; Oxaea austera; Oxaea flavescens; Oxaea flavescens ; Xylocopa brasilianorum; Xylocopa cearenses; Xylocopa frontalis; Xylocopa grisescens; Xylocopa hirsutíssima; Xylocopa nigrocincta; Xylocopa ordinaria; Xylocopa suspecta* |
| **GREAT DEPENDENCE FOR ANIMAL POLLINATION** | |
| **Avocado** (*Persea americana)* | *Frieseomelitta doederleini; Melipona scutellaris; Nannotrigona punctata; Partamona helleri; Tetragonisca angustula; Trigona spinipes* |
| **Guava** *(Psidium guajava)* | *Augochlora esox; Augochloropsis patens; Bombus morio; Centris aenea; Centris inermis; Centris rhodoprocta;Centris tarsata; Centris varia; Epicharis flava; Eulaema nigrita; Exomalopsis auropilosa; Melipona mandacaia; Melipona quadrifasciata; Melipona scutellaris; Melipona subnitida; Nannotrigona punctata; Nannotrigona testaceicornis; Oxaea flavescens; Partamona cupira; Partamona seridoenses; Tetragonisca angustula; Trigona hyalinata; Trigona spinipes; Xylocopa cearenses; Xylocopa frontalis; Xylocopa grisescens* |
| **Sunflower** (*Helianthus annuus)* | *Bombus morio; Frieseomelitta doederleini ; Melipona subnitida; Nannotrigona testaceicornis; Oxaea flavescens; Scaptotrigona bipunctata; Trigona hyalinata; Trigona spinipes; Xylocopa grisescens* |
| **Tomato** *(Lycopersicon esculentum)* | *Bombus morio; Bombus pauloensis; Centris fuscata; Centris nitens; Centris similis; Centris tarsata; Exomalopsis analis; Exomalopsis auropilosa; Melipona fasciculata; Melipona quadrifasciata; Oxaea flavescens; Plebeia droryana; Pseudaugochlora gramínea; Tetragonisca angustula; Tetrapedia diversipes; Xylocopa muscaria* |
| **MODEST DEPENDENCE FOR ANIMAL POLLINATION** | |
| **Coconut** (*Cocos nucifera)* | *Plebeia poecilochroa; Trigona guianae; Trigona spinipes* |
| **Cotton** (*Gossypium hirsutum, G. barbadense, G. arboreum, G. herbaceum)* | *Melissodes nigroaenea; Melissoptila cnecomala; Melissoptila uncicornis; Schwarziana quadripunctata; Xylocopa suspecta; Xylocopa grisescens* |
| **Coffee** *(Coffea arabica, C. canephora, C. liberica)* | *Epicharis flava; Frieseomelitta meadewaldoi; Melipona quadrifasciata; Tetragonisca angustula; Tetragonisca fiebrigi; Trigona amalthea; Trigona fulviventris* |
| **LITTLE DEPENDENCE FOR ANIMAL POLLINATION** | |
| **Bean** (*Phaseolus vulgaris)* | *Bombus morio; Bombus pauloensis; Megachile aetheria; Megachile aureiventris; Megachile tenuitarsis; Thygater analis; Xylocopa augusti; Xylocopa bimaculata; Xylocopa frontalis; Xylocopa nigrocincta* |
| **Mandarin** *(Citrus reticulata)* | *Melipona scutellaris; Tetragonisca angustula; Trigona spinipes* |
| **Persimmon** (*Cavanillea philippensis, Diospyros kaki)* | *Centris fuscata; Euglossa securigera; Xylocopa grisescens* |
