# Supplementary material for: Projected climate change threatens pollinators and crop production in Brazil
Source: PLoS One. 2017 Aug 9;12(8):e0182274. doi: 10.1371/journal.pone.0182274 (PMC5549956; doi:10.1371/journal.pone.0182274)
Supplement: S2 Table — (DOCX) [file pone.0182274.s002.docx]

**S2 Table.** Data sources retrieved from speciesLink and GBIF website

| 1. Bee Biology and Systematics Laboratory |
| --- |
| 1. Coleção Camargo - FFCLRP/USP RPSP |
| 1. Coleção de Abelhas do Museu de Ciências e Tecnologia da PUCRS |
| 1. Coleção de Abelhas MCP |
| 1. Coleção de Entomologia do Laboratório de Biologia Vegetal |
| 1. Coleção de Hymenoptera INPA  INPA-HYMENOPTERA |
| 1. Coleção de Hymenoptera ZUEC-HYM |
| 1. Coleção Entomológica da UFES  UFES-ENTOMOLOGIA |
| 1. Coleção Entomológica de Santa Cruz do Sul  CESC |
| 1. Coleção Entomológica do Depto. de Sistemática e Ecologia  DSEC |
| 1. Coleção Entomológica dos Campos Gerais do Paraná  CECG |
| 1. Coleção Entomológica Moure & Costa CEMEC |
| 1. Coleção Entomológica Paulo Nogueira-Neto - IB/USP CEPANN |
| 1. Coleção Entomológica Pe. Jesus Santiago Moure (Hymenoptera)  DZUP-HYMENOPTERA |
| 1. Collaborative databasing of North American bee collections within a global informatics network project AMNH-BEE |
| 1. Entomological collections Natural History Museum London NHM-LONDON-ENT |
| 1. Entomology Division, Yale Peabody Museum |
| 1. Fototeca Cristiano Menezes FCM |
| 1. Hymenoptera Collection - Instituto Nacional de Pesquisas da Amazônia (INPA) |
| 1. Hymenoptera specimen database of Kyushu University |
| 1. Illinois Natural History Survey INHS Insect Collection INHS-INSECTS |
| 1. Laboratório de Ecologia e Biogeografia de Insetos da Caatinga LEBIC |
| 1. Natural History Museum Rotterdam (NL) - Insecta collection |
| 1. Naturalis Biodiversity Center (NL) - Hymenoptera |
| 1. Snow Entomological Museum Collection KU-SEMC |
