# Supplementary material for: Projected climate change threatens pollinators and crop production in Brazil
Source: PLoS One. 2017 Aug 9;12(8):e0182274. doi: 10.1371/journal.pone.0182274 (PMC5549956; doi:10.1371/journal.pone.0182274)
Supplement: S3 Table — (DOCX) [file pone.0182274.s003.docx]

**S3 Table.** Shifts on pollinators’ occurrence probability for all crops analyzed (13 crops) considering A) the overall average of decrease in probability; B) the number and percentage of municipalities that will potentially face decrease or increase on pollinators’ occurrence probability (total number of municipalities analyzed equals to 4975)

| **A) Decreased on pollinators’ occurrence probability** | | |
| --- | --- | --- |
| Percentage | Standard deviation |  |
| 12.7 | 11.2 |  |
| **B) Municipalities** | | |
| Number of municipalities showing *decrease* on pollinators’ occurrence probability | Percentage | |
| 4369 | 87.8 | |
| Number of municipalities showing *increase* on pollinators’ occurrence probability | Percentage | |
| 606 | 12.2 | |
