# Supplementary material for: Projected climate change threatens pollinators and crop production in Brazil
Source: PLoS One. 2017 Aug 9;12(8):e0182274. doi: 10.1371/journal.pone.0182274 (PMC5549956; doi:10.1371/journal.pone.0182274)
Supplement: S4 Table — We considered 25% of the highest values of negative shift on pollinators and, from those, the 15 municipalities that present the highest percentage of GDP associated to the analyzed crop. Acerola was not included due to the lack of data. (DOCX) [file pone.0182274.s004.docx]

**S4 Table.** Municipalities that will potentially face the highest negative shift on pollinators’ occurrence probability and that present the highest percentage of Gross Domestic Product (GDP) associated to the analyzed crops. We considered 25% of the highest values of negative shift on pollinators and, from those, the 15 municipalities that present the highest percentage of GDP associated to the analyzed crop. Acerola was not included due to the lack of data.

| **Municipality** | **State** | **Crop** | **mean shift on pollinators** | **percent crop / GDP** |
| --- | --- | --- | --- | --- |
| Águas Vermelhas | Minas Gerais | annatto | -0.233 | 0.174 |
| Altinópolis | São Paulo | avocado | -0.202 | 0.186 |
| Alto Alegre | Rio Grande do Sul | tomato | -0.381 | 18.383 |
| Alto Feliz | Rio Grande do Sul | persimmon | -0.052 | 0.159 |
| Alto Taquari | Mato Grosso | cotton | -0.146 | 4.703 |
| Alto Taquari | Mato Grosso | sunflower | -0.268 | 0.035 |
| Amparo | Paraíba | persimmon | -0.421 | 0.469 |
| Araçoiaba | Pernambuco | annatto | -0.235 | 0.091 |
| Araguari | Minas Gerais | sunflower | -0.281 | 0.042 |
| Arapuã | Paraná | bean | -0.293 | 8.824 |
| Assaí | Paraná | persimmon | -0.065 | 0.120 |
| Bandeirantes | Mato Grosso do Sul | cotton | -0.132 | 2.507 |
| Bandeirantes | Mato Grosso do Sul | sunflower | -0.252 | 0.030 |
| Barão | Rio Grande do Sul | avocado | -0.222 | 0.043 |
| Belmonte | Bahia | coconut | -0.116 | 2.610 |
| Belo Vale | Minas Gerais | mandarin | -0.233 | 19.351 |
| Boa Esperança | Paraná | sunflower | -0.320 | 0.073 |
| Bom Jesus da Penha | Minas Gerais | coffee | -0.355 | 33.253 |
| Bom Jesus da Penha | Minas Gerais | sunflower | -0.612 | 0.328 |
| Bom Sucesso do Sul | Paraná | bean | -0.153 | 16.795 |
| Bonfim | Minas Gerais | mandarin | -0.260 | 1.546 |
| Bonfim | Minas Gerais | passionfruit | -0.214 | 1.485 |
| Brejo Grande | Sergipe | coconut | -0.091 | 4.733 |
| Brochier | Rio Grande do Sul | mandarin | -0.307 | 5.320 |
| Brodowski | São Paulo | avocado | -0.213 | 0.099 |
| Brunópolis | Santa Catarina | bean | -0.180 | 9.336 |
| Cabo Verde | Minas Gerais | coffee | -0.311 | 49.941 |
| Caconde | São Paulo | coffee | -0.322 | 32.809 |
| Cajuru | São Paulo | avocado | -0.215 | 0.223 |
| Canavieiras | Bahia | coconut | -0.101 | 5.589 |
| Cândido de Abreu | Paraná | bean | -0.280 | 11.179 |
| Cândido Rodrigues | São Paulo | guava | -0.400 | 0.497 |
| Capetinga | Minas Gerais | coffee | -0.427 | 36.757 |
| Cardoso | São Paulo | tomato | -0.344 | 17.253 |
| Carlópolis | Paraná | guava | -0.245 | 1.321 |
| Carmo do Rio Claro | Minas Gerais | sunflower | -0.484 | 0.066 |
| Casa Branca | São Paulo | mandarin | -0.229 | 2.088 |
| Cascalho Rico | Minas Gerais | passionfruit | -0.313 | 2.613 |
| Cerro Negro | Santa Catarina | bean | -0.214 | 10.766 |
| Chapadão do Céu | Goiás | cotton | -0.129 | 17.640 |
| Chapadão do Sul | Mato Grosso do Sul | cotton | -0.110 | 10.831 |
| Claraval | Minas Gerais | coffee | -0.368 | 35.931 |
| Colorado | Rio Grande do Sul | annatto | -0.192 | 0.282 |
| Conceição da Aparecida | Minas Gerais | coffee | -0.323 | 48.924 |
| Conde | Bahia | coconut | -0.084 | 5.628 |
| Congonhinhas | Paraná | persimmon | -0.058 | 0.150 |
| Coqueiro Baixo | Rio Grande do Sul | avocado | -0.200 | 0.041 |
| Coronel Macedo | São Paulo | bean | -0.145 | 15.739 |
| Coronel Xavier Chaves | Minas Gerais | mandarin | -0.202 | 4.650 |
| Corumbatai do Sul | Paraná | passionfruit | -0.144 | 4.391 |
| Corumbatai do Sul | Paraná | avocado | -0.248 | 0.257 |
| Costa Rica | Mato Grosso do Sul | cotton | -0.106 | 18.768 |
| Cristais Paulista | São Paulo | coffee | -0.409 | 41.584 |
| Cristalina | Goiás | cotton | -0.139 | 3.226 |
| Cruzeiro do Sul | Paraná | annatto | -0.362 | 0.669 |
| Cruzmaltina | Paraná | tomato | -0.473 | 35.761 |
| Curral de Dentro | Minas Gerais | annatto | -0.206 | 0.317 |
| Divinolândia | São Paulo | passionfruit | -0.247 | 1.101 |
| Divisópolis | Minas Gerais | annatto | -0.253 | 0.664 |
| Duartina | São Paulo | persimmon | -0.069 | 0.127 |
| Esplanada | Bahia | passionfruit | -0.199 | 1.158 |
| Felício dos Santos | Minas Gerais | annatto | -0.190 | 1.389 |
| Fernão | São Paulo | passionfruit | -0.205 | 1.565 |
| Fortuna de Minas | Minas Gerais | annatto | -0.306 | 0.150 |
| Frei Lagonegro | Minas Gerais | annatto | -0.287 | 0.143 |
| General Carneiro | Paraná | cotton | -0.120 | 10.092 |
| Godoy Moreira | Paraná | tomato | -0.488 | 20.680 |
| Goianópolis | Goiás | tomato | -0.375 | 18.659 |
| Guarda-Mor | Minas Gerais | cotton | -0.156 | 6.348 |
| Guimarânia | Minas Gerais | passionfruit | -0.233 | 1.090 |
| Harmonia | Rio Grande do Sul | mandarin | -0.247 | 7.051 |
| Inajá | Paraná | tomato | -0.505 | 27.481 |
| Indiaroba | Sergipe | coconut | -0.072 | 7.503 |
| Ipiranga | Paraná | bean | -0.188 | 9.408 |
| Irapuru | São Paulo | passionfruit | -0.126 | 1.697 |
| Itaí | São Paulo | cotton | -0.150 | 6.869 |
| Itamogi | Minas Gerais | coffee | -0.454 | 49.468 |
| Itaperuçu | Paraná | mandarin | -0.244 | 1.796 |
| Itápolis | São Paulo | guava | -0.313 | 0.510 |
| Itarema | Ceará | coconut | -0.129 | 3.421 |
| Ivaí | Paraná | bean | -0.255 | 22.465 |
| Jaborandi | São Paulo | cotton | -0.249 | 64.852 |
| Jandaíra | Rio Grande do Norte | coconut | -0.111 | 38.465 |
| Jandaíra | Bahia | coconut | -0.079 | 21.113 |
| Jandaíra | Bahia | passionfruit | -0.191 | 12.827 |
| Japoatã | Sergipe | coconut | -0.090 | 4.232 |
| Japoatã | Sergipe | passionfruit | -0.244 | 1.374 |
| Jardinópolis | São Paulo | avocado | -0.213 | 1.358 |
| Júlio Mesquita | São Paulo | guava | -0.271 | 0.644 |
| Junqueirópolis | São Paulo | annatto | -0.190 | 0.495 |
| Juruaia | Minas Gerais | coffee | -0.358 | 44.861 |
| Lagarto | Sergipe | passionfruit | -0.175 | 1.842 |
| Lagoa Formosa | Minas Gerais | tomato | -0.453 | 14.740 |
| Lagoa Formosa | Minas Gerais | sunflower | -0.245 | 0.028 |
| Leme | São Paulo | sunflower | -0.356 | 0.023 |
| Lidianópolis | Paraná | guava | -0.213 | 2.148 |
| Lucianópolis | São Paulo | persimmon | -0.059 | 0.782 |
| Maratá | Rio Grande do Sul | mandarin | -0.284 | 2.122 |
| Medeiros | Minas Gerais | bean | -0.187 | 9.361 |
| Monte Alegre de Minas | Minas Gerais | sunflower | -0.248 | 0.027 |
| Monte Santo de Minas | Minas Gerais | coffee | -0.414 | 34.224 |
| Monte Santo de Minas | Minas Gerais | avocado | -0.203 | 0.042 |
| Montenegro | Rio Grande do Sul | mandarin | -0.195 | 1.436 |
| Neópolis | Sergipe | coconut | -0.075 | 5.785 |
| Nepomuceno | Minas Gerais | avocado | -0.203 | 0.062 |
| Nísia Floresta | Rio Grande do Norte | avocado | -0.238 | 0.042 |
| Nova Resende | Minas Gerais | coffee | -0.344 | 61.698 |
| Novo Itacolomi | Paraná | sunflower | -0.340 | 0.055 |
| Onça de Pitangui | Minas Gerais | tomato | -0.530 | 26.838 |
| Ouro Verde de Goiás | Goiás | tomato | -0.384 | 12.324 |
| Pacatuba | Sergipe | coconut | -0.093 | 8.577 |
| Palmeira | Santa Catarina | bean | -0.204 | 37.964 |
| Paranacity | Paraná | annatto | -0.243 | 0.915 |
| Paranapanema | São Paulo | cotton | -0.146 | 5.541 |
| Paranapanema | São Paulo | persimmon | -0.053 | 0.474 |
| Parapuã | São Paulo | passionfruit | -0.139 | 1.204 |
| Passos | Minas Gerais | sunflower | -0.606 | 0.056 |
| Pequi | Minas Gerais | tomato | -0.557 | 15.630 |
| Piaçabuçu | Alagoas | coconut | -0.098 | 4.657 |
| Piedade dos Gerais | Minas Gerais | mandarin | -0.239 | 1.149 |
| Pimenta | Minas Gerais | tomato | -0.547 | 13.797 |
| Pirangi | São Paulo | mandarin | -0.214 | 1.924 |
| Pirangi | São Paulo | guava | -0.430 | 1.023 |
| Poço das Antas | Rio Grande do Sul | avocado | -0.206 | 0.071 |
| Portelândia | Goiás | cotton | -0.104 | 8.767 |
| Pratápolis | Minas Gerais | sunflower | -0.696 | 0.216 |
| Presidente Bernardes | Minas Gerais | tomato | -0.347 | 21.695 |
| Presidente Olegário | Minas Gerais | cotton | -0.111 | 4.895 |
| Prudentópolis | Paraná | bean | -0.281 | 9.753 |
| Reserva | Paraná | bean | -0.235 | 9.867 |
| Ribeirão Corrente | São Paulo | coffee | -0.407 | 33.641 |
| Rio Branco do Sul | Paraná | mandarin | -0.194 | 1.903 |
| Rio Manso | Minas Gerais | avocado | -0.209 | 0.071 |
| Rio Vermelho | Minas Gerais | annatto | -0.261 | 0.191 |
| Romaria | Minas Gerais | coffee | -0.307 | 50.525 |
| Sabinópolis | Minas Gerais | annatto | -0.199 | 0.347 |
| Santa Cruz da Conceição | São Paulo | sunflower | -0.380 | 0.119 |
| Santa Luzia do Itanhy | Sergipe | coconut | -0.077 | 18.108 |
| Santa Luzia do Itanhy | Sergipe | passionfruit | -0.212 | 1.113 |
| Santa Maria | Rio Grande do Norte | avocado | -0.279 | 0.187 |
| Santa Maria | Rio Grande do Norte | persimmon | -0.252 | 0.090 |
| Santa Tereza | Rio Grande do Sul | guava | -0.262 | 0.546 |
| Santa Tereza | Rio Grande do Sul | avocado | -0.215 | 0.229 |
| Santana do Itararé | Paraná | persimmon | -0.069 | 2.169 |
| Santana do Itararé | Paraná | passionfruit | -0.137 | 1.349 |
| Santo Amaro das Brotas | Sergipe | coconut | -0.083 | 2.997 |
| Santo Antônio da Alegria | São Paulo | tomato | -0.578 | 22.382 |
| São Francisco | São Paulo | guava | -0.227 | 0.552 |
| São João Batista do Glória | Minas Gerais | sunflower | -0.509 | 0.020 |
| São João das Duas Pontes | São Paulo | cotton | -0.158 | 2.028 |
| São José da Varginha | Minas Gerais | tomato | -0.551 | 16.462 |
| São José do Hortêncio | Rio Grande do Sul | mandarin | -0.267 | 2.560 |
| São Pedro da União | Minas Gerais | coffee | -0.381 | 53.163 |
| São Pedro da União | Minas Gerais | sunflower | -0.619 | 0.058 |
| São Sebastião da Amoreira | Paraná | avocado | -0.214 | 0.246 |
| São Sebastião da Amoreira | Paraná | persimmon | -0.084 | 0.114 |
| São Simão | São Paulo | mandarin | -0.283 | 1.121 |
| São Tomás de Aquino | Minas Gerais | coffee | -0.442 | 50.537 |
| São Tomé | Rio Grande do Norte | guava | -0.284 | 2.058 |
| Senador Modestino Gonçalves | Minas Gerais | annatto | -0.193 | 1.270 |
| Silvianópolis | Minas Gerais | persimmon | -0.067 | 0.229 |
| Sooretama | Espírito Santo | passionfruit | -0.111 | 1.316 |
| Taiaçu | São Paulo | guava | -0.427 | 2.834 |
| Taiaçu | São Paulo | mandarin | -0.207 | 1.765 |
| Tangará | Rio Grande do Norte | persimmon | -0.276 | 0.252 |
| Taquaritinga | São Paulo | guava | -0.438 | 1.122 |
| Tejupá | São Paulo | bean | -0.152 | 11.941 |
| Terra Roxa | São Paulo | guava | -0.379 | 0.637 |
| Tibagi | Paraná | bean | -0.144 | 13.082 |
| Três Ranchos | Goiás | guava | -0.284 | 2.347 |
| Triunfo | Pernambuco | guava | -0.262 | 1.500 |
| Tupi Paulista | São Paulo | annatto | -0.198 | 0.878 |
| Turvolândia | Minas Gerais | tomato | -0.450 | 31.971 |
| Turvolândia | Minas Gerais | persimmon | -0.063 | 18.236 |
| Una | Bahia | coconut | -0.083 | 3.449 |
| Valinhos | São Paulo | guava | -0.213 | 0.917 |
| Vargem | Santa Catarina | bean | -0.211 | 9.191 |
| Varjão de Minas | Minas Gerais | cotton | -0.142 | 4.017 |
| Vera Cruz | Rio Grande do Norte | persimmon | -0.177 | 0.136 |
| Wenceslau Braz | Paraná | persimmon | -0.056 | 0.162 |
